# Co-Therapy Using Lytic Bacteriophage and Linezolid: Effective Treatment in Eliminating Methicillin Resistant *Staphylococcus aureus* (MRSA) from Diabetic Foot Infections

**DOI:** 10.1371/journal.pone.0056022

**Published:** 2013-02-13

**Authors:** Sanjay Chhibber, Tarsem Kaur, Sandeep Kaur

**Affiliations:** Department of Microbiology, Panjab University, Chandigarh, India; University Hospital Münster, Germany

## Abstract

**Background:**

*Staphylococcus aureus* remains the predominant pathogen in diabetic foot infections and prevalence of methicillin resistant *S.aureus* (MRSA) strains further complicates the situation. The incidence of MRSA in infected foot ulcers is 15–30% and there is an alarming trend for its increase in many countries. Diabetes acts as an immunosuppressive state decreasing the overall immune functioning of body and to worsen the situation, wounds inflicted with drug resistant strains represent a morbid combination in diabetic patients. Foot infections caused by MRSA are associated with an increased risk of amputations, increased hospital stay, increased expenses and higher infection-related mortality. Hence, newer, safer and effective treatment strategies are required for treating MRSA mediated diabetic foot infections. The present study focuses on the use of lytic bacteriophage in combination with linezolid as an effective treatment strategy against foot infection in diabetic population.

**Methodology:**

Acute hindpaw infection with *S.aureus* ATCC 43300 was established in alloxan induced diabetic BALB/c mice. Therapeutic efficacy of a well characterized broad host range lytic bacteriophage, MR-10 was evaluated alone as well as in combination with linezolid in resolving the course of hindpaw foot infection in diabetic mice. The process of wound healing was also investigated.

**Results and Conclusions:**

A single administration of phage exhibited efficacy similar to linezolid in resolving the course of hindpaw infection in diabetic animals. However, combination therapy using both the agents was much more effective in arresting the entire infection process (bacterial load, lesion score, foot myeloperoxidase activity and histopathological analysis). The entire process of tissue healing was also hastened. Use of combined agents has been known to decrease the frequency of emergence of resistant mutants, hence this approach can serve as an effective strategy in treating MRSA mediated foot infections in diabetic individuals who do not respond to conventional antibiotic therapy.

## Introduction

Diabetes is one of the biggest cause of morbidity and mortality worldwide. According to a major international study, an estimated 350 million people in the world have diabetes [1]. Both type 1 and type 2 diabetes lead to hyperglycemia that further results in a number of complications, including damage to nerves (diabetic neuropathy) [2]. Peripheral neuropathy has a central role to play in the development of foot infections. Wounds leading to foot and leg amputation occur in about 30 to 50 percent of patients with diabetes [3].

One of the most common pathogen in acute, previously untreated, superficial infected foot wound in patients with diabetes is *Staphylococcus aureus.* Overuse of antibiotics and the selection of broad- rather than narrow-spectrum agents has contributed towards a high prevalence of methicillin-resistant *S.aureus* (MRSA) in diabetic foot wounds.

MRSA accounts for up to 42.86 % of the *S.aureus* isolates from diabetic foot infections [4]. The prevalence of MRSA in infected foot ulcers is as high as 30% and an increase has been noticed in its incidence in many countries [5]. A recent study from Manchester has reported MRSA isolation in 30.2% of patients, which is a 100% increase as compared to three years earlier [6]. Also MRSA bacteremia in diabetics with foot infections is associated with 43% mortality compared to 20% mortality rate reported with methicillin sensitive *S.aureus* (MSSA) bacteremia [7] The mortality rate is much higher in case of diabetic foot infections caused by MRSA undergoing amputation (43% MRSA vs 9% non-MRSA) [8].

Furthermore, there is evidence that MRSA colonization of chronic ulcers is associated with delayed healing [9], [10]. Strategies to eliminate MRSA from colonized wounds are therefore essential and should include the use of simple, low-cost, effective treatments [10]. The commonly deployed antibiotics to treat MRSA mediated foot infections (tigecycline, vancomycin) are associated with a large number of side effects and *S.aureus* strains resistant to these antibiotics have already emerged in hospital and community settings [11]–[13]. Among the newer drugs, the only drugs specifically approved by US Food and Drug Administration for diabetic foot infections are trovafloxacin (which is now rarely used) and linezolid [14]. Linezolid is a suitable alternative because it has 100% oral bioavailability which allows conversion from intravenous to oral therapies as soon as the patient is clinically stable, thus allowing early discharge and reduced economic burden on the patient. This provides an advantage over comparative therapy (vancomycin and quinupristin/dalfopristin) which can only be delivered parenterally [6].

In addition, a number of clinical studies support the use of linezolid in treating diabetic foot infections caused by MRSA with superior cure rates and excellent tissue penetration into the inflamed soft tissue of diabetic foot infections [15]–[23]. The drug-related adverse events in linezolid treated patients are also generally mild and reversible [24].

An alternative or supplement to antibiotic therapy, which is currently being re-examined, is the use of bacterial viruses (phage/bacteriophage) i.e phage therapy to target bacterial infections particularly refractory to the action of antibiotics. Phage therapy has long been used for treating infections in patients ranging from simple infections such as acne, conjunctivitis, dermatitis, pharyngitis, rhinitis [25], suppurative skin infections [26], [27], surgical and burn wounds [28]–[30] to serious fulminating infections such as deep seated ulcers, osteomyelitis , meningitis and life threatening pneumonia [30], [31]. Moreover, bacteria find it much harder to develop resistance to bacteriophages and there is an abundance of phages in nature with broad host range covering a large number of clinical strains [7]. The ability of a phage to kill and lyse the infecting pathogenic bacteria, their ability to self-replicate and proven safety in various animal models [32]–[39] makes this therapy worth considering especially in immunocompromised individuals such as diabetic patients. Hence, the present study was aimed at isolating a lytic bacteriophage with broad host range and evaluation of its potential for treating MRSA induced hindpaw infection in diabetic mice, administered alone and in combination with the antibiotic.

## Materials and Methods

### Ethical Statement

The experimental protocols were approved by the Institutional Animal Ethics Committee (Approval ID: IAEC/156) of the Panjab University, Chandigarh, India and performed in accordance with the guidelines of Committee for the Purpose of Control and Supervision of Experiments on Animals (CPCSEA), Government of India, on animal experimentation. All efforts were made to minimize the suffering of animals.

### Bacterial Strains

Standard strains of *Staphylococcus aureus* from ATCC, Mannasse, USA were used. These included: *S. aureus* ATCC 43300(MRSA), *S. aureus* ATCC 29213(MSSA), *S. aureus* ATCC 25923(MSSA) and *S.aureus* ATCC 33591(MRSA). Clinical isolates of *S.aureus* (MRSA) procured from Postgraduate Institute of Medical Education and Research (PGIMER), Chandigarh, India were used. These strains were identified on the basis of gram reaction, growth on mannitol salt agar (MSA), catalase activity, and coagulase test. Disk diffusion assay was performed to confirm its resistance towards penicillin, methicillin and oxacillin followed by determination of MIC values against oxacillin by broth microdilution assay as recommended by Clinical and Laboratory Standards Institute (CLSI) [40]. As stated in the CLSI standards, *S.aureus* isolates with oxacillin MICs ≥ 4 µg/ml were taken as methicillin-resistant *S. aureus*(MRSA), and the strains giving MIC values ≤ 4 µg/ml strains as methicillin-sensitive *S. aureus* (MSSA). All such clinical strains giving MIC ≥4 µg/ml were selected , numbered sequentially and stored in 60% glycerol at −80°C and when necessary, maintained on nutrient agar slants at 4°C.

#### Phage isolation, purification and host range determination

The method of Cerveny *et al.* [35] was adopted for the isolation of phages active against *S.aureus* ATCC 43300 from sewage samples. Samples were collected from drainage of different localities in and around Chandigarh. Equal volumes of sewage samples and host bacterium [grown in brain heart infusion broth (BHI) supplemented with 5 mM CaCl_2_] were mixed and incubated overnight at 37°C. After 24 h, the samples were centrifuged, the supernatant filter sterilized and the lysate checked for phage activity using spot assay as described by Chang *et al.* [41]. Phage titration was done using double agar overlay method as described by Adams [42] and phage titers were expressed as plaque forming units (PFU/ml). The phage MR-10 was then tested for its host range against a panel of 34 clinical isolates of *S.aureus* (MRSA) and other ATCC strains by spot test.

#### Phage adsorption rate, one-step growth curve, phage DNA isolation

The adsorption rate of phage MR-10 was determined by the method of Adams [42]. Phage suspension was added at a multiplicity of infection (MOI) of 0.1 to the *S.aureus* 43300 culture and incubated at 37°C. Aliquots were taken at 5 min intervals and the number of free infectious phage particles was calculated by phage titration. A one-step growth curve was performed as described by Chhibber *et al.* [38]. MR-10 phage was added at a MOI of 0.1 to the cells of *S. aureus* 43300 and allowed to adsorb for 24 min at room temperature. The mixture was then centrifuged (10,000 rpm, 10 min, 4°C) and the pellet containing infected cells was suspended in 1 ml of BHI broth with CaCl_2_ (final conc. of 5 mM) followed by incubation at room temperature. Samples were taken periodically in duplicate at 5 min interval for a period of 1 h, immediately diluted and titrated by the double-layer technique.

Bacteriophage DNA was extracted following the standard protocol of Sambrook *et al.* [43] by treating the concentrated phage suspension with proteinase K and sodium dodecyl sulphate followed by extraction with phenol: chloroform. The extracted DNA after precipitation with chilled ethanol was finally dissolved in TE buffer (10 mM Tris-HCl, pH 7.0, 1.0 mM EDTA, pH 7.0). λ/HindIII marker was run in parallel for estimation of molecular size.

#### Transmission electron microscopy

In order to observe phage morphology, transmission electron microscopy (TEM) of the MR-10 phage was performed as described by Goodridge *et al.* [44]. Drops of ultra-centrifuged phage samples (1, 00, 000 g for 2 h, 4°C; L-80, Beckman Instrument, Switzerland) were dropped on nitrocellulose coated grids (diameter, 3 mm; 300 meshes) stained with 2% (w/v) potassium phosphotungustate (pH 6.8–7.2) for 10 sec and examined under a transmission electron microscope (Hitachi H 7500, Tokyo, Japan) at 80 KV.

#### Animals

BALB/c female mice, 4–6 weeks old weighing 20–25 g were used in this study. The animals were obtained from Central Animal House, Panjab University, Chandigarh, India. The animals were kept in polycarbonate cages housed in well aerated rooms with a 12-h light/12-h dark cycle at 25±2°C, fed with standard rodent diet and water ad libitum.

#### Induction of Diabetes in BALB/c mice

Diabetes as per the protocol of Pan *et al.* [45] was induced by giving two intra-peritoneal injections of alloxan monohydrate (150 mg/kg body weight) at 48-hour intervals in mice fasted overnight. One week after the second injection, blood glucose levels (both random as well as fasting) were recorded. The blood sample was obtained by tail clipping method of Oladiende *et al.* [46] and checked by glucose oxidase method using a pre-calibrated Glucometer (One touch Horizon™).

#### Whole blood killing assay

Heparinized blood was collected from the retro-orbital track of diabetic and non-diabetic BALB/c mice. Blood killing assay was performed as per the method of Rich and Lee [47]. Microfuge tubes were labeled and 200 µl of mouse blood from each mouse was mixed with 100 µl of *S.aureus* 43300 to yield a final concentration of 10^5^ CFU/ml. The samples were incubated at 37°C and aliquots were removed after 0,30,60,90 and 120 minutes. Samples were serially diluted and plated on nutrient agar plates to quantify the CFU/ml.

#### Establishment of *S.aureus* 43300 induced hindpaw infection in diabetic mice [47]

*S. aureus* 43300 was cultivated for 24 h at 37°C in brain heart infusion broth. Next day, cells were pelleted and washed twice with phosphate-buffered saline (PBS). Bacterial suspension prepared in PBS was adjusted so as to achieve a cell density corresponding to a range of bacteria inoculums (10^5^,10^6^,10^7^ and 10^8^ CFU/ml). The number of CFU/ml was confirmed by quantitative plate count. Diabetic BALB/c mice were taken and distributed in five different groups of 12 animals (n = 12) each. The mice were anaesthesized by giving *i.p* injection of 100 mg/kg ketamine and 10 mg/kg xylazine. The planter aspect of both the left and right hindpaw of each mice was disinfected with 70% alcohol.10 µl of suspension of *S.aureus* ATCC 43300 was injected at a depth of 2–4 mm into the planter-proximal aspect of the hindpaw. Each of the four groups received different inoculum doses. Animals of the fifth group received same volume of PBS injected into their hindpaws. Two animals from each group were killed on day 1, 3, 5, 7, 9 and 12 post bacterial challenge and both the hindpaws (left and right) of each mice were amputated, defleshed and processed separately. The tissue was homogenized, and dilutions of the homogenates were plated to determine the bacterial burden.

#### Phage protection studies

Therapeutic potential of bacteriophage, MR-10 specific for *S.aureus* 43300 was evaluated for its ability to resolve experimental hindpaw foot infection in diabetic BALB/c mice. The phage was administered locally at a dose of 10^8^ PFU/ml (MOI-100) near the site of inoculation into the hindpaw. Diabetic BALB/c mice were randomly divided into four groups (each group containing 12 mice each).

Group 1: Diabetic mice were infected with *S.aureus* 43300(10^6^ CFU/ml).

Group 2: Diabetic mice were infected with *S.aureus* 43300 followed by administration of phage at a multiplicity of infection (MOI) – 100 [30 minutes post-infection].

Group 3: Diabetic mice were infected with *S.aureus* 43300 followed by administration of linezolid (25 mg/kg/per oral).

Group 4: Diabetic mice were infected with *S.aureus* 43300 followed by administration of phage at a MOI of 100 as well as simultaneous administration of linezolid (25 mg/kg/per oral).

(**Note**: The optimal dose of phage to be used in protection study was based on the results of a preliminary experiment carried out using different MOI and the MOI giving maximum decrease in log CFU was thus selected).

The parameters used to monitor the progress of infection included a) bacterial load in infected and treated hindpaws b) Oedema and Lesion scoring c) Footpaw myeloperoxidase (MPO) levels and d) Histopathological examination.

#### Bacterial load in hindpaw

Bacterial load was assessed as per the method of Park *et al.* [48]. Mice from each group were taken and killed on day 1, 3,5,7,9 and 12 post-infection by cervical dislocation. After disinfection, their hindpaws were amputated, defleshed and homogenized. The tissue homogenates were serially diluted. Bacterial load in terms of CFU/ml was evaluated by plating each dilution on nutrient agar plates and the phage titers were determined (in terms of PFU/ml) by plating the dilution on a lawn of *S. aureus* 43300.

#### Oedema and lesion scoring

Six hours post-inoculation on the same day i.e day 0, and later on day 1, 3,5,7,9 and 12 post-infection, the oedema of the infected hindpaw of all animals was checked with the help of vernier caliper and the lesion was scored on a scale of 0 to 4 as follows:

- 0- No redness, no oedema with thickness of hindpaw in the range of 0.14–0.19 mm.
- 1- Slight redness with slight oedema with thickness of hindpaw in the range of 0.20–0.25 mm.
- 2- Visible redness and moderate oedema with thickness of hindpaw in the range of 0.26–0.30 mm.
- 3- Severe redness and visible oedema with thickness of hindpaw in the range of 0.31–0.36 mm.
- 4- Severe redness and pronounced visible oedema with thickness of hindpaw in the range of 0.37–0.42 mm.

#### Myeloperoxidase (MPO) estimation

Mice from each group (same groups as those categorized for phage protection studies with 12 animals per group) were killed and their hindpaws amputated, defleshed and washed in sterile normal saline. Homogenized foot paw samples were processed for MPO determination as per the method of Greenberger *et al.* [49]. The absorbance was read immediately at 490 nm over a period of 4 minutes. MPO was calculated as the change in optical density (O.D)×dilution factor (D.F).

#### Histopathological examination

Extent of injury caused by *S.aureus* and healing of the infected hindpaw following phage therapy was assessed on the basis of histopathological analysis of the injured and recovered paw following the method of Brans *et al.* [50]. The sections were picked on separate slides, stained with hematoxylin and eosin (Hi-Media, Mumbai) and the slides then examined under a microscope to evaluate the extent of damage.

#### Statistical methods

All data are expressed as mean ± standard deviation of replicated values where indicated. The statistical significance of differences between groups was determined by the Student’s t-test(two groups), one-way ANOVA followed by a Tukey test using Sigma Stat, Graph pad prism (Graph pad software, San Diego, CA). p value of less than 0.01 was considered statistically significant.

## Results

**Figure 1:**
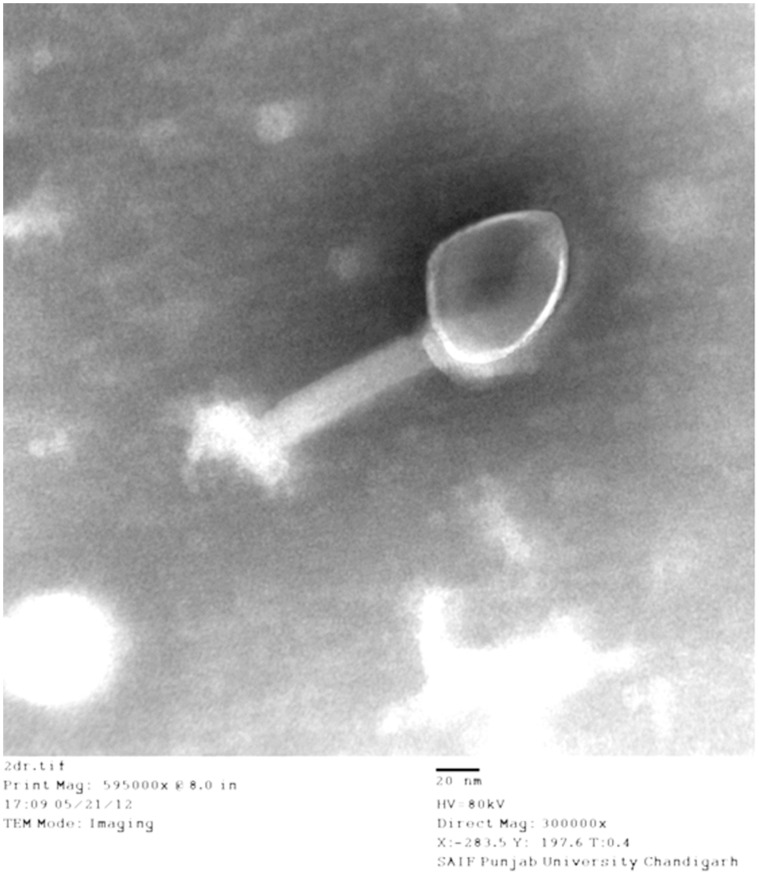
Transmission Electron Micrograph of *S.aureus* specific phage MR-10.

Out of the five isolated phages, MR-10 gave the broad host range as it showed activity on all the four ATCC strains and 32/34 clinical isolates (94%). Therefore, phage MR-10 was selected for further study. The time of adsorption of 99% phage population was calculated to be 24 minutes and the adsorption rate (K) was 0.972×10^−8^ ml/min. One step growth curve revealed that phage MR-10 exhibited a latent period of 15 minutes and burst size of 57 phages per cell. The electron micrographs (Fig. 1) revealed that phage MR-10 had an icosahedral head with a diameter of 56±2.30 nm and contractile tail (120±4.12 nm) separating from the head by a constricted neck region and terminated at a basal tuft. It consisted of dsDNA with a genome size of 24 kb. It belonged to Order *Caudovirales* and Family *Myoviridae.*

### Induction of Diabetes in Mice

The average random blood glucose level of normal BALB/c mice was found to be 139.6±4.16 mg/dl whereas the fasting blood glucose level (after overnight fasting) was 104.6±9.81 mg/dl. Fasting glucose levels were checked daily post-alloxan injection. Mice with fasting blood glucose levels in the range of 150–180 mg/dl, 4–5 days post-injection were termed as moderately diabetic. However, 15 days after alloxan administration, the fasting blood glucose level was found to be 200±6.18 mg/dl whereas the random blood glucose levels were ≥600 mg/dl. Such animals were considered severely diabetic and selected for infection studies.

### Whole Blood Killing Assay

**Figure 2:**
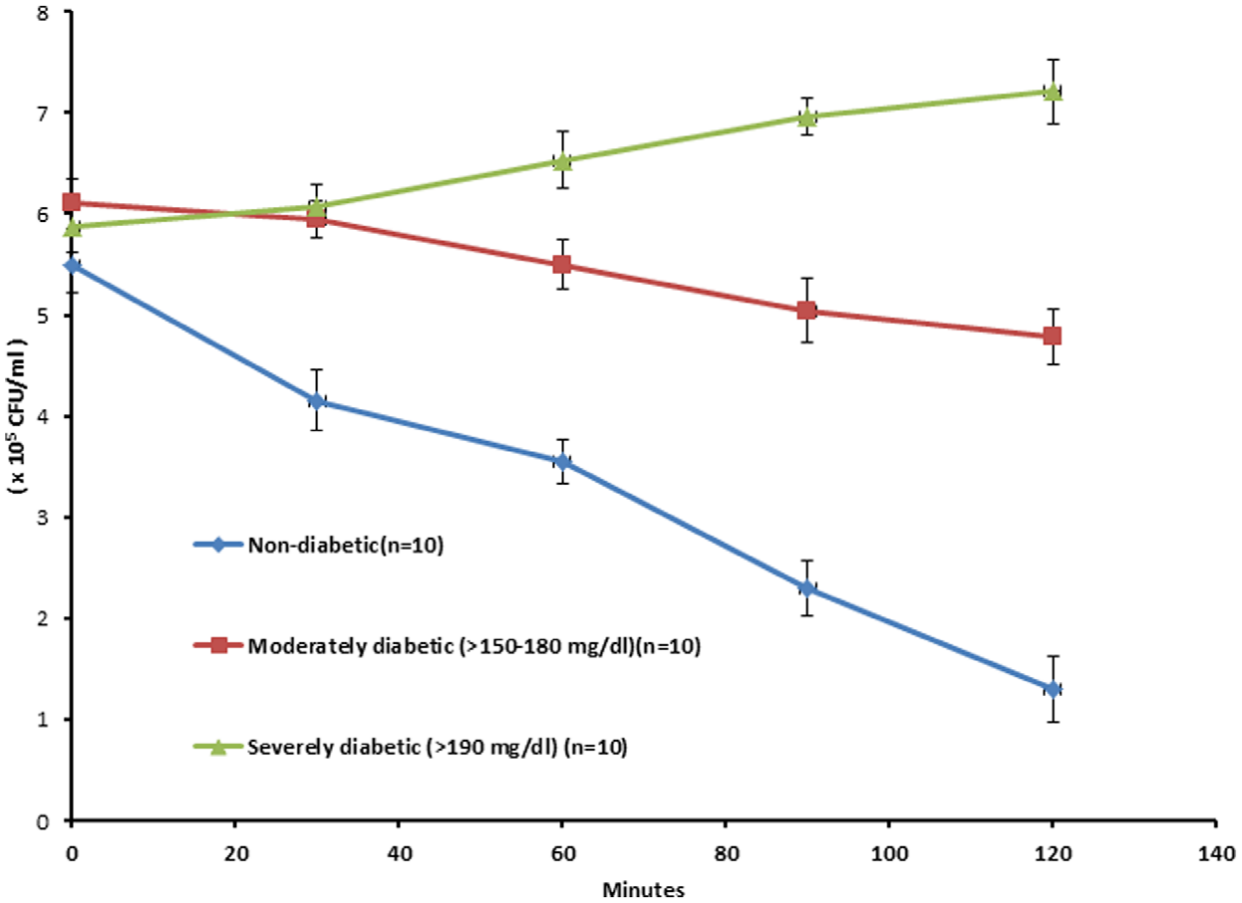
Bacterial load (CFU/ml) as compared to non-diabetic control after incubation of *S.aureus* ATCC 43300 in blood obtained from different groups of animals. [Data points represent mean ±S.D of ten independent values (n = 10)].

The bacterial count of *S.aureus* 43300 when incubated with mouse blood of non-diabetic mice showed a significant decrease from initial 5.5×10^5^±0.25 CFU/ml to a 3.5×10^5^±0.14 CFU/ml after 60 min of contact period. The counts further declined to 1.3×10^5^±0.22 CFU/ml on extended incubation (120 minutes) thus representing 75% killing (% decrease from initial inoculum). However, the mice with moderate diabetes (fasting glucose ≥150–180 mg/dl) showed impaired killing as compared to non-diabetic group. Maximum decrease of 21% from initial bacterial count (decrease from initial 6.1×10^5^±0.24 CFU/ml to 4.78×10^5^±0.17 CFU/ml) was observed after 120 minutes of incubation in the blood. In case of severely diabetic mice with a fasting blood glucose level of ≥ 200 mg/dl, the bacteria were able to multiply in the blood of diabetic animals and showed a steady increase from an initial count of 5.87×10^5^±0.18 CFU/ml to 7.21×10^5^±0.30 CFU/ml which is an increase of 22.6% after 120 minutes (Fig. 2).

### Hindpaw Infection Model

**Figure 3:**
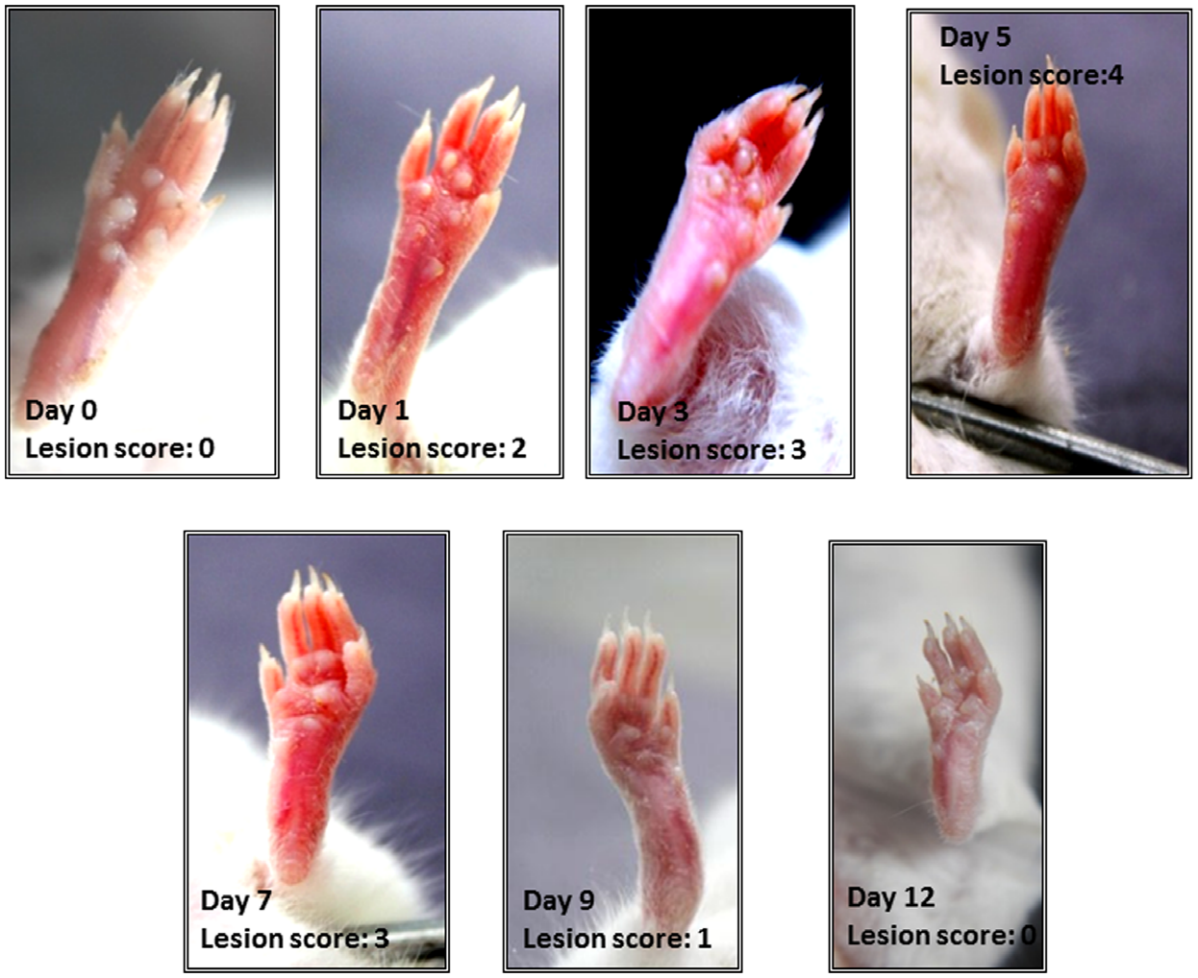
Pictoral representation of *S.aureus* infected hindpaw (10^6^ **CFU/ml along with mean lesion score on different days post infection.**

Since the aim of this study was to access the efficacy of phage on the course of infection process, therefore doses higher than 10^7^ and 10^8^ were not selected as these led to 33.3% mortality in diabetic mice. A dose of 10^6^ CFU/ml was thus chosen as the optimal infectious dose (Table 1) for setting up of significant hindpaw infection in diabetic BALB/c mice. Pictorial representation of *S.aureus* infected hindpaw (10^6^ CFU/ml) and the average lesion score, oedema levels (mm) and bacterial load on different days post infection are depicted in Fig. 3 and Table 1.

**Table 1:** Lesion score, oedema levels (mm) and bacterial load in *S.aureus* infected hindpaw (10^6^ CFU/ml) of mice on different days post infection.

	Day 0 (day of infection)	Day1	Day3	Day5	Day7	Day9	Day12
**Lesion Score**	0	2	3	4	3	1	0
**Oedema(mm)**	0.17±0.01	0.26±0.01	0.34±0.02	0.040±0.02	0.32±0.01	0.24±0.01	0.17±0.01
**Bacterial Load** **(Log CFU/ml)**	*	5.41±0.11	7.89±0.14	5.90±0.08	3.86±0.12	2.01± 0.11	1.01± 0.12

Data is mean±S.D of a minimum of four independent values.

(*): not applicable,

(−): no bacterial counts.

### Phage Protection Study

**Figure 4:**
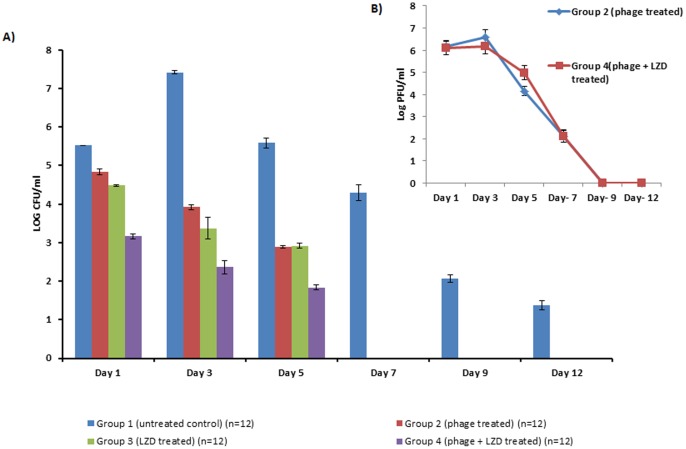
Bacterial load (in terms of Log CFU/ml) in A) Hindpaws of diabetic BALB/c mice following treatment with phage MR-10, linezolid and combination of phageMR-10 and linezolid (25 **mg/kg/per oral) and Phage titers(in terms of Log PFU/ml) in B) Hindpaws of phage treated (group 2 ) and phage + LZD treated (group 4).** [Error bars represent the standard deviation (S.D) from four independent values].

**a) Bacterial load in infected and treated hindpaw.** The results as depicted in Fig. 4A show that when 10^6^ CFU/ml (10^4^ CFU/10 µl) was injected into the hindpaw of untreated control animals (group 1), the bacterial load peaked on day 3 with bacterial burden of 7.42 log CFU/ml, followed by a decline to 5.59 log CFU/ml on day 5 and taking more than 12 days to obtain sterile paws. However, single injection of phage MR-10(10^8^ PFU/ml) (group-2) showed significant reduction (p<0.01) in bacterial load on day 1 itself. The bacterial load was significantly reduced (p<0.01) to 3.92 and 2.89 log CFU/ml respectively on day 3 and 5 which is equivalent to a reduction of 3.5 and 2.7 log cycles as compared to untreated control. Infection was resolved completely by day 7 in group 2 and sterile paws were obtained on day 7. In group 3, animals treated with linezolid also showed a similar pattern with bacterial burden of 3.37 and 2.92 on day 3 and 5 respectively with complete resolution thereafter.

However, maximum reduction in bacterial burden was obtained in group 4 with simultaneous administration of both phage MR-10 and linezolid. The bacterial load reached to 3.16 log CFU/ml on day 1 followed by a steady decline thereafter. On day 3, minimal load of 2.36 log CFU/ml was observed which was a significant reduction of 5 log cycles (p<0.01) in comparison to untreated control that peaked to 7.42 log CFU/ml. Also, bacterial load in hindpaws of animals treated with combination therapy showed comparatively lower burden at all days in comparison to monotherapy group (group 2 and 3). Phage titers (Fig. 4B) initially increased in the hindpaws of animals (group 2 and 4) on day 1 and 3 (due to *in vivo* replication in presence of bacteria) followed by decline on day 5 onwards correlating well with the marked clearance of its host bacteria by day 7 in both the groups.

**b) Oedema and lesion scoring.** As shown in Table 2, an increase in oedema and redness (markers of inflammation in response to infection) was seen in untreated control group which peaked on day 5 with a lesion score of 4. In phage treated mice, an increase in oedema and redness continued till day 5. However, both the oedema and lesion score were significantly less (p<0.01) as compared to untreated control animals on day 3, 5 and 7. Similarly in linezolid (LZD) treated animals, oedema peaked on day 3 with a lesion score of 2 but thereafter it declined with no such signs visible on day 7 onwards. However, in phage + LZD treated group, there was marked reduction (p<0.01) in both redness and oedema as compared to untreated control animals, especially on day 3 and 5 and by day 7, paws were normal. Although lesion scoring based on visual examination of the hindpaw of animals receiving both the agents was almost similar to the lesion score of animals receiving either phage or LZD alone, yet a significant difference (p<0.01) was observed in the oedema level in both the groups on day 3 and 5.

**Table 2:** Lesion score with oedema values on different days post-infection in diabetic mice treated with phage MR-10, linezolid (LZD) and combination of both.

	Untreated Control		Phage MR-10 Treated		LZD treated (25 mg/kg/per oral)		Phage MR-10 + LZD treated	
Days	Lesion score	Oedema (mm)	Lesion score	Oedema (mm)	Lesion score	Oedema (mm)	Lesion score	Oedema (mm)
Day 0	0	0.16±0.01	0	0.17±0.01	0	0.17±0.02	0	0.18±0.02
Day 1	1	0.25±0.02	1	0.22±0.02	1	0.24±0.02	1	0.22±0.02
Day 3	3	0.35±0.02	1	0.28±0.01	2	0.3±0.02	2	0.25±0.02
Day 5	4	0.40±0.01	2	0.26±0.02	1	0.25±0.04	1	0.21±0.02
Day 7	3	0.32±0.02	1	0.2±0.01	0	0.18±0.01	0	0.17±0.01
Day 9	1	0.21±0.01	0	0.18±0.02	0	0.18±0.02	0	0.16±0.01
Day 12	0	0.17±0.01	0	0.17±0.01	0	0.16±0.02	0	0.17±0.01

Data is mean±S.D of a minimum of four independent values.

**Figure 5:**
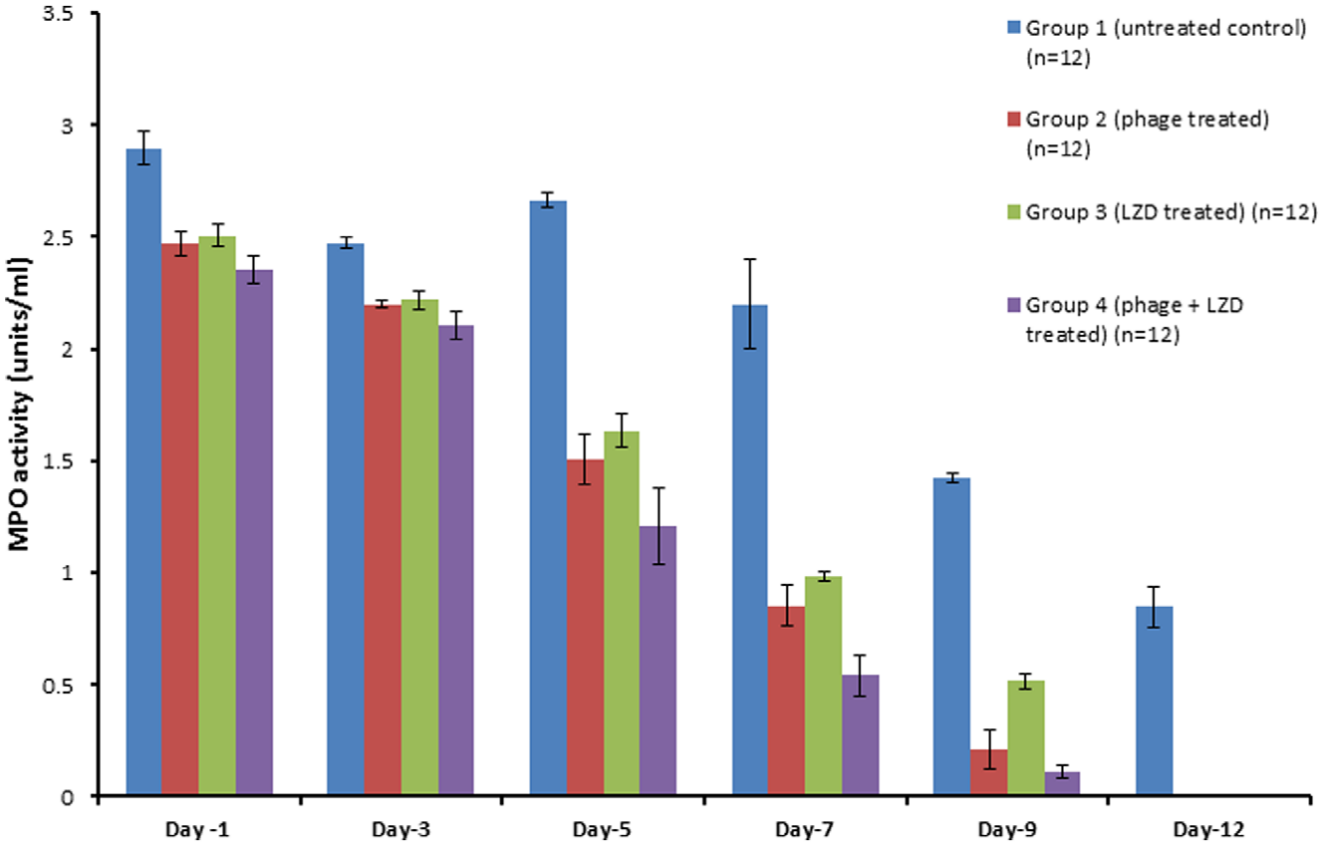
Comparison of MPO levels in the hindpaw of phage MR-10, linezolid and combination of both treated diabetic BALB/c mice at different time intervals. [Error bars represent the standard deviation (S.D) from four independent values].

**c) Myeloperoxidase (MPO) activity.** As seen in Fig. 5, MPO activity in infected hindpaws was significantly less in all the three treatment groups (group 2, 3 and 4) compared to untreated control animals (p<0.01). In the groups 2 and 3, there was a significant reduction in the MPO activity on all the days as compared to untreated control animals (p<0.01) with minimal activity obtained on day 7 and 10. However, marked reduction in tissue MPO activity was seen 5^th^ day onwards in animals treated with combination therapy (group 4). The tissue MPO values of group 4 were significantly reduced (p<0.01) as compared to untreated control animals. The MPO values was also significantly less (p<0.01) than MPO values obtained in group 2 and 3 on day 3, 5 and 9.

**Figure 6:**
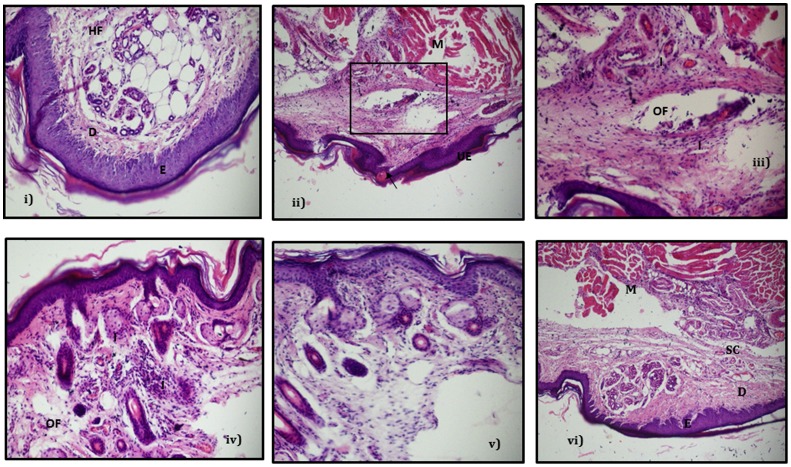
Histopathological examination of infected and treated hindpaws of diabetic mice. Photo micrograph of i) Normal skin showing intact epithelial layers(E,D,M) sebaceous glands and intact hair follicles(HF) (H and E 200X) ii) Skin tissue from hindpaw of infected diabetic mice showing fairly heavy inflammation(I) involving the dermis and extending deep into muscle (H and E 100X) iii) Magnified view of the area (denoted in the rectangular panel in (ii) showing heavy infiltration of polymorphoneutrophils (I) and collection of oedematous fluid in the subcutaneous areas iv) Skin tissue from hindpaw of infected diabetic mice treated with phage MR-10 alone showing moderate inflammation and infiltration (I) is visible with oedema (OF) seen in some areas v) Skin tissue from hindpaw of infected diabetic mice treated with linezolid showing mild inflammatory areas and vi) Skin tissue from hindpaw of infected diabetic mice treated with phage MR-10 and linezolid (in combination) showing normal skin layers with significant reduction in inflammation. (**E**-epidermis, **D**-dermis, **SC**-subcutaneous layer, **M**-muscle layer, **OF**-oedema fluid accumulation, **UE**-ulcerated epridermis, **arrow**- abscess in epidermal layer, **I** –infiltrating neutrophils).

**d) Histopathological examination.** The histopathological section of hindpaw tissue from all the groups were taken on the 5^th^ day post-infection. The control skin showed all the intact layers, namely epidermis with keratin layers, dermis with hair follicles being visible and the fat and muscle layer (Fig. 6). However, when the histopathological sections of infected hindpaw of diabetic mice were observed, the epidermal layer was highly ulcerated and pus containing abscesses in both the dermis and subcutaneous soft tissue were visible. These mice had a dense infiltration of neutrophils and heavy inflammation was seen in the skin, involving the dermis and extending deep into the muscle [Fig. 6(ii)]. In the phage treated group, the skin showed mild infiltration of lymphocytes and the fibroblastic cells in the dermis beneath the epidermis. Although the epidermis appeared normal and non-ulcerated, yet there was moderate inflammation observed in the dermis beneath the epidermis with oedema fluid also visible in the dermal layer [Fig. 6(iv)]. This was indicative of the process of tissue healing which correlated well with the lesion score of 2.0 observed in these animals on day 5. In the linezolid treated group, the epidermis was normal accompanied with only mild inflammatory cells in some areas in the dermis. However, in the animals administered with both phage MR-10 and linezolid, the epidermis was normal, similar to control skin with no signs of ulceration or oedema in any layer with all the skin layers being intact and normal. There was a marked reduction in infiltrating cells with only a small focus of inflammatory cells visible in the dermis [Fig. 6(vi)].

## Discussion

*Staphylococcus aureus* plays a prominent role as an etiological agent of foot infections in diabetic patients. The condition worsens further if the infection is caused by MRSA due to its innate and acquired resistance to many antimicrobials increasing the duration of hospital stay, cost of management and additional risks of morbidity and mortality. It has been estimated that at least 50% of all deaths caused by diabetic foot are the result of infections that are untreatable and caused by resistant strains. The present study reinforces the view that phage therapy as an alternative treatment option for MRSA infections in diabetic patients is worth exploring. Limited work is available on the potential use of phages in treating MRSA infections in diabetic animals [51], [52]. However, none of the workers have studied the effectiveness of phages as part of combination therapy in treating foot infections, that are one of the leading causes of amputation and life long disability among diabetic population. The therapeutic efficacy of characterized phage MR-10 (with a broad host range, belonging to Myoviridae family) in treating *S.aureus* mediated localized foot infection in diabetic mice was evaluated.

Neutrophils from diabetic patients have been shown to exhibit impaired bactericidal activity [53]–[57]. To determine whether leukocytes from diabetic BALB/c mice would show a similar defect, we performed *S.aureus* killing assays with mouse blood. The degree of bactericidal activity in the blood from individual diabetic BALB/c mice in this study correlated well with their blood glucose level. Blood from moderately diabetic mice (fasting glucose ≥150–180 mg/dl) showed poor killing activity of 21% after 120 minutes of incubation. On the contrary, *S. aureus* multiplied in the blood obtained from severly diabetic mice with a fasting blood glucose level of ≥200 mg/dl. In support of these results is the observation of Gresham *et al.* [58] who demonstrated that survival of *S.aureus* inside polymorphoneutrophils contributes towards the pathogenesis of staphylococcal infection in a murine peritonitis model. These findings are also in line with the findings of Rich and Lee [47] who demonstrated poor killing activity of leukocytes obtained from diabetic NOD mice.

Several *in vitro* functional defects of the immune system have been correlated with the metabolic control of diabetic patients. The insulinopenia-induced enzymatic defects which are responsible for inhibiting energy-requiring functions essential for normal functioning of phagocytes and lymphocytes are one of them. This is likely to hamper the normal bactericidal killing efficiency of neutrophils of diabetic patients [59].

The diabetic mice infected with *S.aureus* 43300 were treated with phage MR-10. The results showed that a single injection of 10^8^ PFU/ml was able to resolve the infectious process within a period of 5 days, unlike 15 days required to obtain sterile paws in untreated animals. A significant reduction of ∼3.5 log cycles and 2.7 log cycles in bacterial load was also obtained as compared to control animals on day 3 and 5 (p<0.01). This correlated well with the lesion scoring and oedema levels . This finding corroborates the results of an earlier study where in rats infected with *S.aureus* MRSA CSV-36 in an excision wound model and administered with *S. aureus* phage Ø SH-56 showed sterile skin abscess after 6 days [52]. In the present study, phage MR-10 prevented initial multiplication of the pathogen since a bacterial count of 4.84 log CFU /ml was obtained after 24 hours of infection which was almost similar to the bacterial inoculum injected (10^4^ CFU/10 µl). However, later phage multiplied (as evident by the phage titers that peaked on day 3) and was able to effectively arrest the entire infection within the next 48–72 hours. In addition, the process of tissue healing was hastened before the establishment of full fledged infection in diabetic animals.

Linezolid is one of the latest drugs recommended for treating diabetic foot infections [23]. It has activity against many of the gram-positive organisms and is available in both intravenous and oral formulations, making it convenient for use in both inpatient and outpatient settings. In the group that received linezolid (25 mg/kg) orally, bacterial load was significantly reduced in the first 24 hours and by day 5 the bacterial load was significantly reduced to 2.92 log CFU/ml (p<0.01). On day 7 post-infection, the bacterial load was negligible and infection was completely resolved. The oedema and lesion score also correlated well with the decreased bacterial load. On comparing these results with those obtained with phage MR-10, it was observed that both the agents (phage and linezolid) when administered alone 30 minutes after infection showed almost similar efficacy in resolving MRSA induced hindpaw infection in diabetic mice.

In animals receiving combination therapy (10^8^ PFU/ml, given directly into the hindpaw along with linezolid administered orally) the infection process was well- controlled with significant reduction of ∼5 log cycles in bacterial load as compared to untreated mice (p<0.01). This reduction in bacterial load was maximum in comparison to when phage or linezolid were given alone. However, compared to differences observed in microbial load at the infection site in the combination therapy group, no marked differences were seen in the lesion scores of this group compared to animals receiving LZD only. This discrepancy probably was due to the visual scoring of the lesions as it was based entirely on the development of redness and erythema around the infected hindpaw. The oedema was measured by using vernier caliper and a range of measurements were clubbed in one lesion score. Though in combination therapy the oedema levels were significantly less than those observed with monotherapy on days 3 and 5 yet these were given the same score as the values fell within that range. However, the entire process of infection and tissue injury was shorter and milder as it healed faster than in animals given either phage or linezolid alone. This emphasizes that phage given along with linezolid effectively controlled the pathogen population by their combined action. Linezolid being a bacteriostatic antibiotic can easily stop further growth of *S.aureus* population which can then be easily cleared off by the lytic phage and thus prevent the initial establishment of infection. This combination therapy has its own advantages. Not only it was effective in controlling the infection process, but it has also been reported in literature that use of phage and antibiotic together also checks the development of resistant mutants as the two agents differ in their mechanism of action [60]. Besides this, the maximum decrease in tissue MPO levels was also seen in this group of mice that received combined therapy with minimal MPO activity obtained on day 5 and 7. This could be due to the fact that combined therapy effectively contained the pathogen and once the growth of invading bacteria was arrested at the site of injury, neutrophil accumulation declined and this correlated well with tissue healing. The results of histopathological examination of control (untreated) and treated skin also substantiated these observations.

From these observations it is concluded that phage MR-10 with a broad host range (lytic toward MRSA as well as MSSA strains) showed efficacy similar to a potent currently used antibiotic i.e linezolid. A combination therapy using bacteriophage and linezolid was found to be more effective in controlling the entire process of hindpaw infection in diabetic mice as compared to antibiotic or phage given alone. Since emergence of linezolid resistant strains has already been reported therefore, combination of two agents will always help in decreasing the development of resistant mutants. Hence, co-therapy using phage and linezolid can take care of the critical problem of resistance in modern medicine, particularly in immuno-suppressed patients. Phage therapy therefore presents a new window and reinforces the view that it can act as an alternative treatment option for MRSA infections in diabetic patients.

## References

[1] Danaei G, Finucane MM, Lu Y, Singh GM, Cowan MJ, et al (2011) National, regional, and global trends in fasting plasma glucose and diabetes prevalence since 1980: systematic analysis of health examination surveys and epidemiological studies with 370 country-years and 2.7 million participants. The Lancet 378(9785): 31–40. 10.1016/S0140-6736(11)60679-X 21705069

[2] Umpierrez GE, Scott D, Isaac SD, Bazargan N, You X, et al (2002) Hyperglycemia: An Independent Marker of In-Hospital Mortality in Patients with Undiagnosed Diabetes. Journal of Clinical Endrocrinology and Metabolism 87: 978–982. 10.1210/jcem.87.3.8341 11889147

[3] Bader MS. Diabetic foot infection (2008) Am. Fam. Physician 78(1): 71–9. 18649613

[4] Murugans S, Mani KR, UmaDevi P (2008) Prevalence of methicillin resistant *Staphylococcus aureus* among diabetes patients with foot ulcers and their antimicrobial susceptibility pattern. Journal of Clinical and Diagnostic Research 2: 979–984.

[5] Rogers LC, Bevilacqua NJ (2009) MRSA in Diabetic Foot. Podiatry Management. (http://www.podiatrym.com/cme/Nov09CME1.pdf).

[6] Turner JM, Hakeem LM, Lockman KA, Bhattacharyya DN, Campbell IW (2004) Diabetic MRSA foot infection – role of linezolid therapy. British Journal of Diabetes and Vascular Disease 4: 44.

[7] Talon D, Woronoff-Lemsi MC, Limat S (2002) The impact of resistance to methicillin in *Staphylococcus aureus* bacteremia on mortality. Eur J Intern Med 13(1): 31–36. 11836080 10.1016/s0953-6205(01)00189-3

[8] Grimble SA, Magee TR, Galland RB (2001) Methicillin resistant *Staphylococcus aureus* in patients undergoing major amputation. Eur J Vasc Endovasc Surg 22(3): 215–218. 11506513 10.1053/ejvs.2001.1436

[9] Dang CN, Prasad YD, Boulton AJ, Jude EB (2003) Methicillin-resistant *Staphylococcus aureus* in the diabetic foot clinic: a worsening problem. Diabetes and Medicine 20: 159–161. 10.1046/j.1464-5491.2003.00860.x 12581269

[10] Bowling FL, Salgami EV, Boulton AJ (2007) Larval therapy: a novel treatment in eliminating methicillin-resistant *Staphylococcus aureus* from diabetic foot ulcers. Diabetes Care 30: 370–371. 17259512 10.2337/dc06-2348

[11] Rybak MJ, Albrecht LM, Boike SC, Chandrasekar PH (1990) Nephrotoxicity of vancomycin alone and with an aminoglycoside. Journal of Antimicrobial Chemotherapy 25: 679–687. 2351627 10.1093/jac/25.4.679

[12] Gordon RJ, Lowy FD (2008) Pathogenesis of methicillin‐resistant *Staphylococcus aureus* infection. Clinical Infectious Diseases 46 (S5): S350–S359. 10.1086/533591 PMC2474459 18462090

[13] Stein GE, Craig WA (2006) Tigecycline: a critical analysis. Clinical Infectious Diseases 43: 518–524. 16838243 10.1086/505494

[14] Lipsky BA (2004) A report from the international consensus on diagnosing and treating the infected diabetic foot. *Diabetes Metab Res Rev* 20: S68–S77. 15150818 10.1002/dmrr.453

[15] Lipsky BA, Itani K, Norden C (2004) Treating foot infections in diabetic patients: a randomized, multicenter, open-label trial of linezolid versus ampicillin-sulbactam/co-amoxiclav. Clinical Infectious Diseases 38: 17–24. 14679443 10.1086/380449

[16] Itani KM, Weigelt J, Li JZ, Duttagupta S (2005) Linezolid reduces length of stay and duration of intravenous treatment compared with vancomycin for complicated skin and soft tissue infections due to suspected or proven methicillin-resistant *Staphylococcus aureus* (MRSA). International Journal of Antimicrobial Agents 26: 442–448. 16289514 10.1016/j.ijantimicag.2005.09.003

[17] Sharpe JN, Shively EH, Polk HC (2005) Clinical and economic outcomes of oral linezolid *versus* intravenous vancomycin in the treatment of MRSA-complicated, lower extremity skin and soft tissue infections caused by methicillin-resistant *Staphyloccus aureus* . American Journal of Surgergy 189: 425–428. 10.1016/j.amjsurg.2005.01.011 15820454

[18] Weigelt JH, Kaafarani M, Itani KM, Swanson RN (2004) Linezolid eradicates MRSA better than vancomycin from surgical-site infections. American Journal of Surgery 188: 760–766. 15619496 10.1016/j.amjsurg.2004.08.045

[19] Weigelt J, Itani K, Stevens D, Lau W, Dryden M, et al (2005) Linezolid versus vancomycin in treatment of complicated skin and soft tissue infections. Antimicrobial Agents and Chemotherapy 49: 2260–2266. 15917519 10.1128/AAC.49.6.2260-2266.2005 PMC1140485

[20] Stein GE (2007) Schooley S, Peloquin CA, Missavage A, Havlichek DH (2007) Linezolid tissue penetration and serum activity against strains of methicillin-resistant *Staphylococcus aureus* with reduced vancomycin susceptibility in diabetic patients with foot infections. Journal of Antimicrobial and Chemotherapy 60: 819–823. 10.1093/jac/dkm271 17673476

[21] Falagas ME, Siempos II, Vardakas KZ (2008) Linezolid versus glycopeptide or beta -lactam for treatment of Gram-positive bacterial infections: meta-analysis of randomised controlled trials. Lancet Infectious Diseases 8: 53–66. 18156089 10.1016/S1473-3099(07)70312-2

[22] Dryden MS (2011) Linezolid pharmacokinetics and pharmacodynamics in clinical treatment. Journal of Antimicrobial Chemotherapy 66 Suppl 4iv7–iv15. 21521707 10.1093/jac/dkr072

[23] Wiskirchen DE, Shepard A, Kuti JL, Nicolau DP (2011) Determination of Tissue Penetration and Pharmacokinetics of Linezolid in patients with diabetic foot infections using in vivo microdialysis. Antimicrobial Agents and Chemotherapy 55(9): 4170–4175. 21709078 10.1128/AAC.00445-11 PMC3165366

[24] Lipsky BA, Berendt AR, Deery HG, Embil JM, Joseph WS, et al (2004) Diagnosis and treatment of diabetic foot infections. Clinical Infectious Diseases 39: 885–910. 15472838 10.1086/424846

[25] Sakandelidze VM (1991) The combined use of specific phages and antibiotics in different infectious allergoses. Vrach Delo 3: 60–63. 2042352

[26] Cislo M, Dabrowski M, Weber-Dabrowska B, Woyton A (1987) Bacteriophage treatment of suppurative skin infections. Archivum Immunologiae et Therapiae Experimentalis 2: 175–183. 3447533

[27] Weber-Dabrowska B, Dabrowski M, Slopek S (1987) Studies on bacteriophage penetration in patients subjected to phage therapy. Archivum Immunologiae et Therapiae Experimentalis 35: 563–568. 3332066

[28] Kochetkova VA, Mamontov AS, Moskovtseva RL, Erastova EI, Trofimov EI, et al (1989) Phagotherapy of postoperative suppurative-inflammatory complications in patients with neoplasms. Sov Med 16: 23–26. 2799488

[29] Lazareva EB, Smirnov SV, Khvatov VB, Spiridonove TG, Bitkova EE, et al (2001) Efficacy of bacteriophage in complex treatment of patients with burn trauma. Antibiotiki i khimioterapiya 46(1): 10–14. 11221078

[30] Mathur MD, Bidhani S, Mehndiratta PL (2003) Bacteriophage therapy: an alternative to conventional antibiotics. J. Assoc. Physicians India 51: 593–596. 15266928

[31] Stroj L, Weber-Dabrowska B, Partyka K, Mulczyk M, Wojcik M (1993) Successful treatment with bacteriophage in purulent cerebrospinal meningitis in a newborn. Neurologia I neurochirurgia polska 3: 693–698. 10540729

[32] Bogovazova GG, Voroshilova NN, Bondarenko VM (1991) The efficacy of *Klebsiella pneumoniae* bacteriophage in the therapy of experimental *Klebsiella* infection. Zhurnal Mikrobiologii Epidemiologii Immunobiologii 4: 5–8. 1882608

[33] Biswas B, Adhya S, Washart P, Paul B, Trostel AN, et al (2002) Bacteriophage therapy rescues mice bacteremia from a clinical isolate of vancomycin-resistant *Enterococcus faecium* . Infection and Immunity 70: 204–210. 11748184 10.1128/IAI.70.1.204-210.2002 PMC127648

[34] Bull JJ, Levin BR, DeRouin T, Walker N, Bloch CA (2002) Dynamics of success and failure in phage and antibiotic therapy in experimental infections. BMC Microbiology 2: 35. 12453306 10.1186/1471-2180-2-35 PMC138797

[35] Cerveny KE, DePaola A, Duckworth DH, Gulig PA (2002) Phage therapy of local and systemic disease caused by *Vibrio vulnificus* in iron - dextran -treated mice. Infection and Immunology 70(11): 6251–6262. 10.1128/IAI.70.11.6251-6262.2002 PMC130292 12379704

[36] Duckworth D, Gulig P (2002) Bacteriophage: potential treatment for bacterial infections. Biodrugs 16: 57–62. 11909002 10.2165/00063030-200216010-00006

[37] Capparelli R, Parlato M, Borriello G, Salvatore P, Iannelli D (2007) Experimental phage therapy against *Staphylococcus aureus* in mice. Antimicrobial Agents and Chemotherapy 51: 2765–2773. 17517843 10.1128/AAC.01513-06 PMC1932491

[38] Chhibber S, Kaur S, Kumari S (2008) Therapeutic potential of bacteriophage in treating *Klebsiella pneumoniae* B5055-mediated lobar pneumonia in mice. Journal of Medical Microbiology 57: 1508–1513. 19018021 10.1099/jmm.0.2008/002873-0

[39] Kumari S, Harjai K, Chhibber S (2009) Efficacy of bacteriophage treatment in murine burn wound infection induced by *Klebsiella pneumoniae* . Journal of Microbiology and Biotechnology 19(6): 622–628. 19597322 10.4014/jmb.0808.493

[40] Wayne PA (2006) Methods for dilution antimicrobial susceptibility tests for bacteria that grow aerobically. Approved standard M7-A7. Clinical and Laboratory Standards Institute.

[41] Chang HC, Chen CR, Lin JW, Shen GH, Chang KM, et al (2005) Isolation and characterization of novel giant *Stenotrophomonas maltophilia* phage ФSMA5. Applied and Environmental Microbiology 71: 1387–1393. 10.1128/AEM.71.3.1387-1393.2005 PMC1065149 15746341

[42] Adam MH (1959) Bacteriophages, Interscience Publishers, New York, N.Y. 450–456.

[43] Sambrook J, Fritsch EF, Maniatis T (1989) Molecular cloning: a laboratory manual, 2nd ed. Cold Spring Habor laboratory Press, Cold Spring Harbor, N.Y.

[44] Goodridge L, Gallacci A, Griffiths MW (2003) Morphological, host range and genetic characterization of two coliphages. Applied and Environmental Microbiology 69(9): 5364–5371. 12957924 10.1128/AEM.69.9.5364-5371.2003 PMC194992

[45] Pan D, Liu H (2008) Preventive effect of ordinary and hyperimmune bovine colostrums on mice diabetes induced by alloxan. African Journal of Biotechnology 7 (24): 4369–4375.

[46] Oladeinde FO, Kinyua AM, Laditan AA, Michelin R, Bryant JL, et al (2007) Effect of *Cnidoscolus aconitifoliu*s leaf extract on the blood glucose and insulin levels of inbred type 2 diabetic mice. Cellular and Molecular Biology 53(3): 34–41. 17531147

[47] Rich J, Lee JC (2005) The pathogenesis of *Staphylococcus aureus* infection in the diabetic NOD mouse. Diabetes 54: 2904–2910. 16186391 10.2337/diabetes.54.10.2904

[48] Park S, Rich J, Hanses F, Lee JC (2009) Defects in innate immunity predispose C57BL/6J-Leprdb/Leprdb mice to infection by *Staphylococcus aureus* . Infection and Immunity 77: 1008–1014. 19103772 10.1128/IAI.00976-08 PMC2643648

[49] Greenberger MJ, Strieter RM, Kunkel SL, Danforth JM, Laichalk LL, et al (1996) Neutralization of Macrophage inflammatory protein-2 attenuates neutrophil recruitment and bacterial clearance in murine *Klebsiella* pneumonia. Journal of Infectious Diseases 173: 159–165. 8537653 10.1093/infdis/173.1.159

[50] Brans TA, Dutrieux RP, Hoekstra MJ, Kreis RW, Du Pont JS (1994) Histopathological evaluation of scalds and contact burns in the pig model. Burns 20: 548. 10.1016/0305-4179(94)90090-6 8198744

[51] Sunagar R, Patil SA, Chandrakanth RK (2010) Bacteriophage therapy for *Staphylococcus aureus* bacteremia in streptozotocin-induced diabetic mice. Research in Microbiology 161(10): 854–860. 20868746 10.1016/j.resmic.2010.09.011

[52] VinodKumar CS, Srinivasa H, Basavarajappa KG, Patil U, Bandekar N, et al (2011) Abrogation of *Staphylococcus aureus* wound infection by bacteriophage in diabetic rats. International Journal of Pharmaceutical Sciences and Drug Research 3(3): 202–207.

[53] Tan JS, Anderson JL, Watanakunakorn C, Phair JP (1975) Neutrophil dysfunction in diabetes mellitus. Journal of Laboratory Clinical Medicine 85: 26–33. 1141727

[54] Repine JE, Clawson CC, Goetz FC (1980) Bactericidal function of neutrophils from patients with acute bacterial infections and from diabetics Journal of Infectious Diseases. 142: 869–875. 10.1093/infdis/142.6.869 7007524

[55] Wilson RM, Reeves WG (1986) Neutrophil phagocytosis and killing in insulin-dependent diabetes. Clinical and Experimental Immunology 63: 478–484. 3084140 PMC1577392

[56] Tater D, Tepaut B, Bercovici JP, Youinou P (1987) Polymorphonuclear cell derangements in type I diabetes. Horm Metab Res 19: 642–647. 3440569 10.1055/s-2007-1011899

[57] Marhoffer W, Stein M, Schleinkofer L, Federlin K (1993) Evidence of ex vivo and in vitro impaired neutrophil oxidative burst and phagocytic capacity in type 1 diabetes mellitus. Diabetes Res Clin Pract 19: 183–188. 8319516 10.1016/0168-8227(93)90112-i

[58] Gresham HD, Lowrance JH, Caver TE, Wilson BS, Cheung AL (2000) Survival of *Staphylococcus aureus* inside neutrophils contributes to infection. Journal of Immunology 164: 3713–3722. 10.4049/jimmunol.164.7.3713 10725730

[59] Moutschen MP, Scheen AJ, Lefebvre PJ (1992) Impaired immune responses in diabetes mellitus: analysis of the factors and mechanisms involved. Relevance to the increased susceptibility of diabetic patients to specific infections. Diabetes and Metabolism 18(3): 187–201. 1397473

[60] Bedi MS, Verma V, Chhibber S (2009) Amoxicillin and specific bacteriophage can be used together for eradication of biofilm of *Klebsiella pneumoniae* B5055. World Journal of Microbiology and Biotechnology 25 1145–1151.

